# Non-invasive detection of bilirubin concentrations during the first week of life in a low-resource setting along the Thailand–Myanmar border

**DOI:** 10.1136/bmjpo-2024-002754

**Published:** 2024-09-28

**Authors:** Germana Bancone, Mary Ellen Gilder, Elsie Win, Gornpan Gornsawun, Paw Khu Moo, Laypaw Archasuksan, Nan San Wai, Sylverine Win, Borimas Hanboonkunupakarn, Francois Nosten, Verena Ilona Carrara, Rose McGready

**Affiliations:** 1 Shoklo Malaria Research Unit, Mahidol-Oxford Tropical Medicine Research Unit, Faculty of Tropical Medicine, Mahidol University, Mae Sot, Thailand; 2 Centre for Tropical Medicine and Global Health, Nuffield Department of Medicine, University of Oxford, Oxford, UK; 3 Mahidol-Oxford Tropical Medicine Research Unit (MORU), Faculty of Tropical Medicine, Mahidol University, Bangkok, Thailand; 4 Department of Clinical Tropical Medicine, Faculty of Tropical Medicine, Mahidol University, Bangkok, Thailand; 5 Institute of Global Health, Faculty of Medicine, University of Geneva, Geneve, Switzerland

**Keywords:** Jaundice, Neonatology, Low and Middle Income Countries

## Abstract

**Background:**

Neonatal hyperbilirubinaemia (NH) is a common problem worldwide and is a cause of morbidity and mortality especially in low-resource settings.

**Methods:**

A study was carried out at Shoklo Malaria Research Unit (SMRU) clinics along the Thailand–Myanmar border to evaluate a non-invasive test for diagnosis of NH in a low-resource setting. Performance of a transcutaneous bilirubinometer Dräger Jaundice Meter JM-105 was assessed against routine capillary serum bilirubin testing (with BR-501 microbilirubinometer) before phototherapy during neonatal care in the first week of life. Results were analysed by direct agreement and by various bilirubin thresholds used in clinical practice. Total serum bilirubin was also measured in cord blood at birth and tested for prediction of hyperbilirubinaemia requiring phototherapy in the first week of life.

**Results:**

Between April 2020 and May 2023, 742 neonates born at SMRU facilities were included in the study. A total of 695 neonates provided one to nine capillary blood samples for analysis of serum bilirubin (total 1244 tests) during the first week of life. Performance of transcutaneous bilirubinometer was assessed in 307 neonates who provided 687 paired transcutaneous capillary blood tests. Bilirubin levels were also measured in 738 cord blood samples. Adjusted values of transcutaneous bilirubinometer showed excellent agreement with capillary serum bilirubin concentration (intraclass correlation coefficient=0.923) and high sensitivity (>98%) at all clinical thresholds analysed across 3 years of sampling and multiple users. Concentrations of bilirubin detected in cord blood were not useful in identifying neonates at risk of hyperbilirubinaemia requiring treatment.

**Conclusions:**

The transcutaneous bilirubinometer is a reliable tool to screen neonates and identify those needing confirmatory blood testing. Bilirubin concentrations in cord blood are not predictive of hyperbilirubinaemia in neonates.

**WHAT IS ALREADY KNOWN ON THIS TOPIC**

- Non-invasive detection of bilirubin concentrations in cord blood and transcutaneously can support better clinical care of neonates at risk of hyperbilirubinaemia, especially in low-resource settings.
- The performance of transcutaneous bilirubinometers in dark-skinned neonates is not well established.

**WHAT THIS STUDY ADDS**

- This study was the first carried out in neonates of Karen and Burman ethnicity born at the Thailand–Myanmar border.
- The study provides new data on the good performance of the transcutaneous bilirubinometer used by locally trained birth attendants.
- The results show that cord blood bilirubin levels are not predictive of the risk of hyperbilirubinaemia in the first days of life.

**HOW THIS STUDY MIGHT AFFECT RESEARCH, PRACTICE OR POLICY**

- This study adds to the growing body of knowledge on the performance and utility of non-invasive screening tools and diagnostics to improve neonatal health in low-resource settings and low-income and middle-income countries.

## Introduction

 Physiological jaundice is common in newborns and usually resolves spontaneously within 2 weeks. However, when unconjugated bilirubin blood exceeds an age-dependent concentration (resulting in neonatal hyperbilirubinaemia, NH), it can cross the blood–brain barrier and lead to serious complications, including neurological damage and death.

Several risk factors are associated with neonatal jaundice, including inherited glucose-6-phosphate dehydrogenase (G6PD) deficiency, ABO and Rhesus incompatibility, prematurity, prolonged labour, traumatic birth and infections.^1^ Standard of care should include identification of at-risk neonates and monitoring of bilirubin levels before discharge from the birthing centre. Treatment involves blue light phototherapy and in some cases exchange transfusion based on bilirubin nomograms adjusted by gestational age and the neonate’s age.^2^

Newborns in low-income and middle-income countries (LMIC) often have a late diagnosis of NH.^3^ Major barriers to appropriate postnatal jaundice care are uncertainty of gestational age, lack of diagnostic tools for risk factors and for bilirubin levels, and poor access to treatment (especially for the most severe cases requiring exchange transfusion).

Total and unconjugated bilirubin can be measured by high-performance liquid chromatography, which is the gold standard test; however, most commonly, total serum bilirubin (TSB) alone is measured by more widely available biochemistry analysers or by microbilirubinometers on a venous or capillary blood sample.

Non-invasive approaches have been developed to reduce neonatal pain.^4^ Transcutaneous bilirubinometer (TcB) is a non-invasive screening tool that can detect subcutaneous concentrations of bilirubin by colorimetry from the forehead or sternum and inform the need for confirmatory blood testing for diagnosis of NH and its treatment. TcB has been increasingly used worldwide, with varying reports of its performance according to the age of the neonate, colour of the skin and levels of TSB. In Asia, validation studies for TcB have been carried out in India, Thailand, Malaysia, Japan, Mongolia and China,^5^,^10^ showing promising results.

Bilirubin levels measured non-invasively in cord blood have been tested for prediction of NH developing during the first 72 hours of life^11^,^13^ and might be used as a very early risk predictor of NH,^14^ with the obvious advantage of not requiring an invasive heel prick.

In this study, the performance of TcB was assessed in paired non-invasive transcutaneous measures against TSB detected in capillary blood samples in a population of neonates of Karen and Burman ethnicity born in Shoklo Malaria Research Unit (SMRU) clinics along the Thailand–Myanmar border. Non-invasive cord blood bilirubin concentrations were analysed as a predictor of NH in the first week of life and as a potential screening tool to reduce neonatal skin pricks.

## Materials and methods

The study was conducted at SMRU clinics situated along the Thailand–Myanmar border (Thailand), where free antenatal care and birthing services are provided for migrant women of predominantly Karen and Burman ethnicities. A previous epidemiological birth cohort study in this population^15 16^ identified a high rate of NH (22%) in this community, with a high prevalence of G6PD deficiency (allelic frequency 11.6%) and UGT1A1*6 mutation (allelic frequency 17.0%). There was a low rate of Rh incompatibility (0.2%).

Pregnant women attending SMRU clinics at Wang Pha (WPA) and Maw Ker Thai (MKT) were informed about the study during their antenatal care visits in the third trimester. Informed consent was obtained before labour, and eligibility of neonates was assessed immediately after delivery. Neonates born at an estimated gestational age (EGA) of ≥35 weeks by ultrasound, with no severe maternal complications at delivery and no severe neonatal illnesses, were included.

Two millilitres of blood were collected into EDTA from the umbilical cord and an aliquot was used for TSB measurement. After birth, capillary blood TSB test was performed at routine predischarge check (at 48±12 hours of life) and, whenever clinically indicated, up to 1 week of life. Indication for starting phototherapy treatment followed the recommendations of the UK National Institute for Health and Care Excellence (NICE) guidelines^2^ based on capillary blood TSB testing and using EGA and age-adjusted nomograms. TcB was performed whenever possible, for example, if the neonate was not crying and the TcB device was available; TcB tests were always performed shortly before blood sampling for TSB (maximum 5 min) and TcB results were available immediately (blinded to TSB results). Follow-up in the study was considered completed if a neonate developed NH or was admitted to the special care baby unit (SCBU). TSB measurement continued during and after phototherapy for clinical purposes only while collection of TcB measure stopped.

TSB in cord blood and capillary blood was measured by clinical laboratory technicians. An aliquot of blood was transferred into a heparinised capillary tube (70 μL), centrifuged at 10 000 revolutions per minute (rpm) for 3 min and analysed by a dual-wavelength BR-501 microbilirubinometer (Apel, Japan), according to the manufacturer’s instructions. Quality control (QC) for the microbilirubinometer was run daily, calibration was carried out weekly and external maintenance was performed yearly. The BR-501 bilirubinometer had ±5% accuracy within the measurement range (0–30 mg/dL or 0–513 μmol/L), as reported by the manufacturer.

TcB measures were done using Jaundice Meter JM-105 (Dräger, Germany). The device was used in the MKT clinic in 2020 and in the WPA clinic from 2021 to 2023. Manufacturer’s instructions were followed when performing daily QC procedures. Bilirubin concentrations were measured by placing the device on the neonate’s midsternum in three consecutive readings, with the automatic average used as the final measure. The personnel collecting blood samples and measuring TcB were locally trained skilled birth attendants who follow a standardised SMRU curriculum.^17^ Training on the use of TcB was carried out twice at each clinic before the start of the study; full Standard Operating Procedure (SOP) and simplified visual aid were available during the study.

### Sample size

For the diagnostic performance of TcB compared with the TSB reference test, the sample size was calculated considering estimation of the limits of agreement in Bland-Altman plot, whereby a minimum of 350 individuals is needed to obtain 95% CI of the limits of agreement within a 0.2 SD difference.^18^

### Statistical analyses

Mean and SD were reported for continuous variables. Categorical variables were compared by χ^2^ test and analysis of variance. Bland-Altman plot was used to inspect correspondence between TSB in capillary blood and TcB; intraclass correlation coefficient (ICC) was calculated for assessment of absolute agreement between paired capillary TSB and TcB values using a two-way mixed effects model. Diagnostic performance of TcB (sensitivity, specificity, positive predictive value and negative predictive value) was calculated for the following capillary TSB thresholds: ≥100 μmol/L, ≥150 μmol/L and 25 μmol/L increments, until ≥300 μmol/L.

The area under the curve (AUC) of the receiver operating characteristic (ROC) curve^19^ was calculated for (1) the whole cohort, (2) for neonates followed up for at least 48 hours and (3) at different cord blood TSB thresholds to analyse the risk of NH requiring treatment. For this analysis, neonates’ gestational age was categorised as <38 weeks and ≥38 weeks.^16^

Data were analysed using SPSS V.27 and statistical significance was assessed at the 5% level.

### Patient and public involvement

At the outset of the study, the research team engaged the local population through the Tak Province Community Advisory Board in Thailand. This group comprised community leaders who were asked to advise on study design, process and outcomes of interest, and subsequently approved the study.

## Results

**Figure 1:**
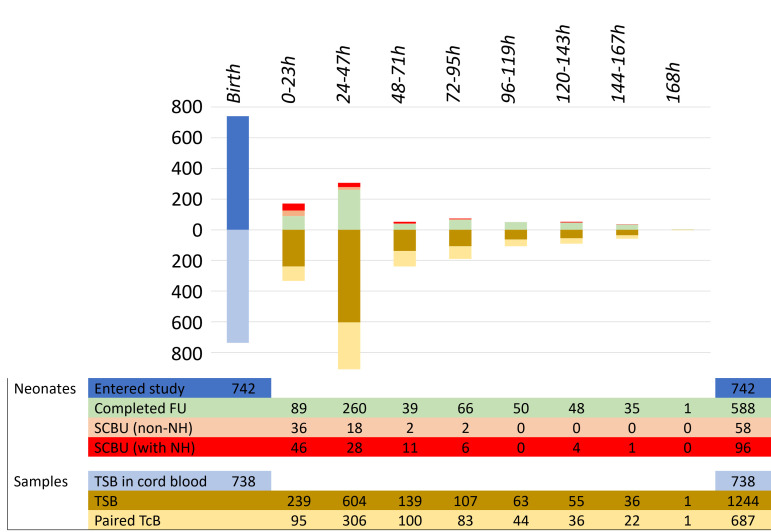
Participants and samples. FU, follow-up; NH, neonatal hyperbilirubinaemia; SCBU, special care baby unit; TcB, transcutaneous bilirubinometer; TSB, total serum bilirubin.

Between April 2020 and May 2023, a total of 742 neonates born at SMRU facilities (477 in MKT and 265 in WPA) entered the study and provided cord blood samples, of which 738 had a valid TSB measurement (figure 1). The mean (SD) EGA in the cohort was 39.3 (1.0) weeks. 49 neonates (6.6%) were born with EGA of 35–<38 weeks and 49.6% of the neonates were female.

Within the first 48 hours, 47 neonates with cord blood TSB results exited the study without a capillary TSB or TcB measurement; of these 47 neonates, 43 were admitted to SCBU, 2 were referred to other hospitals and 2 were discharged. In the following 120 hours, the remaining 695 neonates provided one to nine additional capillary blood samples for analysis of TSB at a mean age of 47 hours, for a total of 1244 analysable samples. Over half of the capillary TSB data point (687 of 1244 from 307 neonates, 55.2%) had a paired TcB value.

During the first 7 days of life, admission to SCBU was recorded in a total of 154 neonates (43 within the first 48 hours and 111 up to 158 hours of life) mostly for early-onset neonatal sepsis and/or NH; phototherapy was recorded in 96 neonates (12.9%), 3 of whom were treated twice. Around half of the neonates (358 of 742) had their last bilirubin checked on the second or third day of life before discharge from the clinic; around 30% had TSB done at follow-up between 4 and 7 days of life.

### Transcutaneous bilirubin versus capillary TSB during the first week of life

A total of 1250 capillary TSB tests results were collected (718 in MKT and 532 in WPA); six data points were excluded from analysis (online supplemental figure S1), for a total of 1244 usable samples.

**Figure 2:**
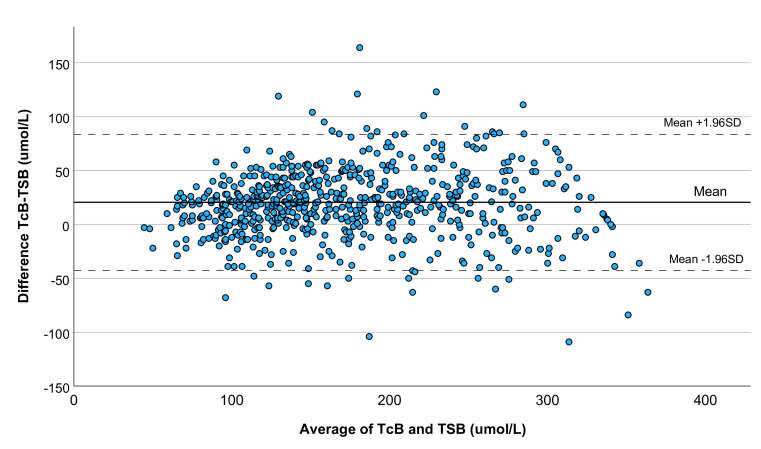
Bland-Altman plot of bilirubin concentration assessed by TcB as compared with TSB. TcB, transcutaneous bilirubinometer; TSB, total serum bilirubin.

A total of 687 paired TcB–capillary TSB tests from 307 neonates were available in the MKT clinic (448 tests) and in the WPA clinic (239 tests). Paired measures were collected between 2 and 168 hours of life. The mean difference (±1.96 SD) in bilirubin levels between TcB and capillary TSB was 20.5 μmol/L (limits of agreement −42.7 to 83.6 μmol/L), as shown in the Bland-Altman plot in figure 2. The analysis included multiple tests performed in the same individuals; slightly wider limits of agreement were obtained when calculated separately for consecutive TcB–capillary TSB repeat (online supplemental table S1).

ICC showed excellent agreement between paired TcB–capillary TSB tests overall (0.923, 95% CI 0.818 to 0.958, p<0.01) and across neonates’ ages (table 1).

**Table 1:** Intraclass correlation coefficient (ICC) for paired TcB–capillary TSB tests across neonates’ ages

Age	0–23 hours	24–47 hours	48–71 hours	72–95 hours	96–119 hours	120–143 hours	144–167 hours	168 hours
N tests	95	306	100	83	44	36	22	1
ICC	0.847	0.788	0.781	0.861	0.945	0.898	0.812	Not applicable
95% CI	0.759 to 0.902	0.399 to 0.879	0.384 to 0.897	0.660 to 0.931	0.895 to 0.971	0.800 to 0.948	0.496 to 0.926	Not applicable

95% CI95% Confidence IntervalICCintraclass correlation coefficientTcBtranscutaneous bilirubinometerTSBtotal serum bilirubin

The performance of TcB, calculated as sensitivity and specificity in identifying capillary TSB levels over different thresholds, improved when 20 μmol/L were added to the reading from TcB (online supplemental table S2), especially at higher thresholds. This improved safety in identifying neonates with higher TSB concentrations at the expense of test specificity.

The results showed a sensitivity of over 98% at all the thresholds analysed, while specificity was usually lower at lower thresholds (100–150 μmol/L), as compared with higher thresholds (250–300 μmol/L), and declined slightly over time. TcB’s overall performance and by clinic when using the adjusted (TcB +20) values, together with the percentage of blood confirmatory tests that would be prevented by using TcB, is presented in online supplemental table S3. The performance of TcB over time is reported in online supplemental table S4.

In a small sample of neonates (n=26), five or more paired longitudinal TcB–capillary TSB tests were available and their consistent time course of bilirubin concentrations is shown in online supplemental figure S2.

### TSB in cord blood for prediction of NH

Among all 738 neonates with cord blood TSB analysable data, the mean (SD) cord blood TSB level was 30.8 (11.7) μmol/L (online supplemental figure S3). They were higher in neonates born with EGA of 35–<38 weeks (34.4 μmol/L) compared with neonates born with EGA of ≥38 weeks (30.5 μmol/L; p<0.01).

**Figure 3:**
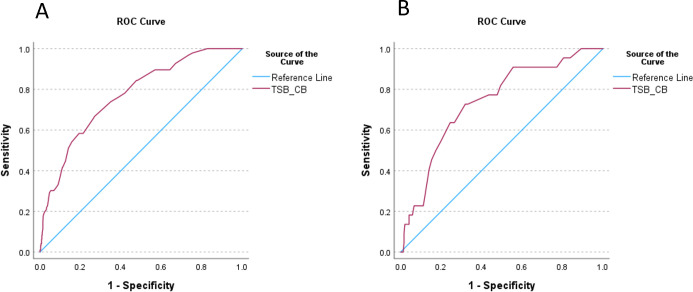
ROC curves for cord blood bilirubin levels predicting NH: (A) whole cohort (N=738) and (B) only neonates followed up for at least 48 hours (n=263). CB, cord blood; NH, neonatal hyperbilirubinaemia; ROC, receiver operating characteristic; TSB, total serum bilirubin.

Statistically higher mean (SD) cord TSB (41.0 (17.6) μmol/L) was observed in the 96 neonates who required phototherapy during the first week of life as compared with neonates who did not (29.1 (9.5) μmol/L; p<0.001). The AUC of the ROC curve was 0.771 (95% CI 0.722 to 0.819) for the whole cohort and 0.738 (95% CI 0.634 to 0.842) when including only neonates followed up for at least 48 hours (n=263) (figure 3). ROC analysis could not identify the thresholds of cord blood TSB that had high sensitivity and specificity in predicting hyperbilirubinaemia and need for phototherapy during the follow-up period (online supplemental table S5).

## Discussion

The current study evaluated non-invasive screening tools to improve clinical care of neonates in a low-resource setting. The population attending SMRU clinics is composed mostly of migrants who live far away from health centres and for whom follow-up visits after birth are not often possible; current practice for hyperbilirubinaemia care in SMRU is based on locally developed guidelines for identification of risk factors and includes a routine predischarge capillary TSB sample at around 48 hours of the neonates’ life. This approach is meant to identify neonates most at risk of developing severe hyperbilirubinaemia during the first week of life, before they leave the birthing centre. Use of TcB in this context could be introduced for the predischarge screen to reduce the number of neonates needing a blood sample (preventing from 2% to over 80% of blood tests depending on the TSB threshold for treatment; online supplemental table S3) or to facilitate painless repeated tests before and during phototherapy treatment. In these scenarios, the screening test would need to show excellent sensitivity in identifying levels of TSB that would require a confirmatory blood testing.

Transcutaneous bilirubin assessments are routinely used in many Western countries and in hospital settings of some tropical countries. Their reliability in populations with more pigmented skin has not been tested widely^20^,^22^ and was evaluated against capillary TSB levels in neonates born along the Thailand–Myanmar border for the first time. The results from this study indicate that TcB values, with an appropriate simple correction to increase sensitivity, can be used reliably against TSB nomograms to identify neonates who need confirmatory laboratory blood tests to detect clinically significant bilirubin levels. Measurements taken by multiple different users over 3 years (March 2020–May 2023) in neonates born in two different clinics, with different EGA and aged 2 hours up to 6 days of life, showed TcB’s very good performance in real-life conditions.

The mean difference against capillary TSB was in line with accuracy data provided by the manufacturer (±25.5 μmol/L in neonates >35 weeks’ gestation) and the ICC was excellent. Performance at different TSB thresholds^23^ showed very good sensitivity, indicating that screening with TcB can correctly identify neonates with higher TSB values who would need a confirmatory blood TSB test. Even with the low specificity observed, especially at 100 μmol/L and 150 μmol/L TSB thresholds (which would translate into performing unnecessary blood tests in neonates with true levels of bilirubin lower than these thresholds), the use of TcB would decrease the total number of invasive blood testing required as compared with current practice at SMRU. Routine use of TcB could also promote more frequent non-invasive assessment of bilirubin concentrations to identify dangerously steep raises in bilirubin levels associated with a more severe course of NH.^2 24^ Use of TcB might be extended to more remote settings as the Dräger JM-105 requires minimal maintenance and no consumables. However, use of devices in field conditions and their performance with long-term use in extreme temperature/humidity conditions would have to be tested (especially their battery life). The price (around US$7000) represents an initial insurmountable investment for most settings with constrained resources, but other transcutaneous devices might be as accurate and more affordable.^25^ Other cheaper screening tools have been developed, such as icterometers^26 27^ and phone-based apps,^28^,^30^ and when properly evaluated could be used by health workers at birthing centres and in remote settings, and even by parents^31^ after discharge from the hospital.

This analysis of bilirubin concentrations in cord blood showed higher mean TSB in premature neonates and in neonates who needed phototherapy during the first week of life, but found no meaningful thresholds for identification of neonates at risk of NH in all neonates or in the subgroup of term neonates. These findings contrast with previously published results^11 32^ but confirm the recommendation from NICE guidelines not to use cord blood bilirubin concentrations to predict hyperbilirubinaemia.^2^

Limitations of the study include not testing the neonates during phototherapy where higher TSB values are observed; this indication is now included in the TcB test specifications (recently reviewed by Ten Kate *et al*^25^). The use of the corrected TcB value, while necessary in a setting with high risk of NH and lack of access to health, might limit its validity; however, the wide international diaspora from resettlement and migration of populations from this border area makes the data useful outside this context. Usability of the device by locally trained clinical staff was not formally assessed; however, during discussions with the midwives involved, the device was deemed easy to use. Finally, the device was not serviced every 12 months as per the manufacturer’s instructions due to the COVID-19 pandemic and this might have had an impact on performance over time. Maintaining multiple technologies and providing appropriate Quality Assurance and QC in low-resource settings can be problematic.

## Conclusions

Most deaths and disabilities from NH occur in LMIC, and this loss of healthy life years is preventable with proper identification and treatment. Together with improved education, there are still measures that can be implemented to improve care for neonatal jaundice in LMIC.^33^ Better and affordable diagnostics are needed globally,^34 35^ and especially in low-resource and conflict areas where clinical care is often dysfunctional. While screening of bilirubin in cord blood in this population does not appear to provide benefit in identifying most at-risk neonates, TcB can be used reliably as a screening tool to inform the need for confirmatory blood testing, supporting more frequent pain-free bilirubin checks and informing better clinical care for neonates in LMIC.

## supplementary material

10.1136/bmjpo-2024-002754 online supplemental file 1

## Data Availability

Data are available upon reasonable request.

## References

[1] Lauer BJ, Spector ND (2011). Hyperbilirubinemia in the newborn. Pediatr Rev.

[2] Health NCCfWsCs (2010). Neonatal jaundice: clinical guideline.

[3] Olusanya BO, Kaplan M, Hansen TWR (2018). Neonatal hyperbilirubinaemia: a global perspective. Lancet Child Adolesc Health.

[4] Witt N, Coynor S, Edwards C (2016). A Guide to Pain Assessment and Management in the Neonate. Curr Emerg Hosp Med Rep.

[5] Akahira-Azuma M, Yonemoto N, Ganzorig B (2013). Validation of a transcutaneous bilirubin meter in Mongolian neonates: comparison with total serum bilirubin. BMC Pediatr.

[6] Boo NY, Ishak S (2007). Prediction of severe hyperbilirubinaemia using the Bilicheck transcutaneous bilirubinometer. J Paediatr Child Health.

[7] Kitsommart R, Pornladnun P, Chomchai C (2013). Accuracy and precision of transcutaneous bilirubinometry in postdischarge Asian neonates. Eur J Pediatr.

[8] Kosarat S, Khuwuthyakorn V (2013). Accuracy of transcutaneous bilirubin measurement in term newborns. J Med Assoc Thai.

[9] Murli L, Thukral A, Sankar MJ (2017). Reliability of transcutaneous bilirubinometry from shielded skin in neonates receiving phototherapy: a prospective cohort study. J Perinatol.

[10] Yamana K, Morioka I, Kurokawa D (2017). Evaluation of BiliCare™ transcutaneous bilirubin device in Japanese newborns. Pediatr Int.

[11] Aktas S, Dogan C, Okmen ZH (2019). Is Cord Blood Bilirubin Level a Reliable Predictor for Developing Significant Hyperbilirubinemia?. Am J Perinatol.

[12] Guan H, Li H, Luo J (2017). Early predictive value of cord blood bilirubin and dynamic monitoring of transcutaneous bilirubin for hyperbilirubinemia of newborns. Saudi J Biol Sci.

[13] Jones KDJ, Grossman SE, Kumaranayakam D (2017). Umbilical cord bilirubin as a predictor of neonatal jaundice: a retrospective cohort study. BMC Pediatr.

[14] Castillo A, Grogan TR, Wegrzyn GH (2018). Umbilical cord blood bilirubins, gestational age, and maternal race predict neonatal hyperbilirubinemia. PLoS One.

[15] Bancone G, Gornsawun G, Peerawaranun P (2022). Contribution of genetic factors to high rates of neonatal hyperbilirubinaemia on the Thailand-Myanmar border. *PLOS Glob Public Health*.

[16] Thielemans L, Peerawaranun P, Mukaka M (2021). High levels of pathological jaundice in the first 24 hours and neonatal hyperbilirubinaemia in an epidemiological cohort study on the Thailand-Myanmar border. PLoS One.

[17] White AL, Min TH, Gross MM (2016). Accelerated Training of Skilled Birth Attendants in a Marginalized Population on the Thai-Myanmar Border: A Multiple Methods Program Evaluation. PLoS One.

[18] Bland JM, Altman DG (1986). Statistical methods for assessing agreement between two methods of clinical measurement. Lancet.

[19] Kumar R, Indrayan A (2011). Receiver operating characteristic (ROC) curve for medical researchers. Indian Pediatr.

[20] Kumar D, Kumar D (2022). A Prospective Comparison of Serum and Transcutaneous Bilirubin in Indian Neonates. J Pediatr Intensive Care.

[21] Olusanya BO, Mabogunje CA, Imosemi DO (2017). Transcutaneous bilirubin nomograms in African neonates. PLoS One.

[22] Yasuda S, Suzuki H, Htun Y (2020). Hour-specific nomogram for transcutaneous Bilirubin in newborns in Myanmar. Pediatr Int.

[23] Okwundu CI, Olowoyeye A, Uthman OA (2023). Transcutaneous bilirubinometry versus total serum bilirubin measurement for newborns. Cochrane Database Syst Rev.

[24] Suzuki H, Yasuda S, Htun Y (2022). Transcutaneous bilirubin-based screening reduces the need for blood exchange transfusion in Myanmar newborns: A single-center, retrospective study. Front Pediatr.

[25] Ten Kate L, van Oorschot T, Woolderink J (2023). Transcutaneous Bilirubin Accuracy Before, During, and After Phototherapy: A Meta-Analysis. Pediatrics.

[26] Olusanya BO, Slusher TM, Imosemi DO (2017). Maternal detection of neonatal jaundice during birth hospitalization using a novel two-color icterometer. PLoS One.

[27] Lee AC, Folger LV, Rahman M (2019). A Novel Icterometer for Hyperbilirubinemia Screening in Low-Resource Settings. Pediatrics.

[28] Aune A, Vartdal G, Jimenez Diaz G (2023). Iterative Development, Validation, and Certification of a Smartphone System to Assess Neonatal Jaundice: Development and Usability Study. *JMIR Pediatr Parent*.

[29] Enweronu-Laryea C, Leung T, Outlaw F (2022). Validating a Sclera-Based Smartphone Application for Screening Jaundiced Newborns in Ghana. Pediatrics.

[30] Taylor JA, Stout JW, de Greef L (2017). Use of a Smartphone App to Assess Neonatal Jaundice. Pediatrics.

[31] Xue G, Zhang H, Ding X (2023). Parental detection of neonatal jaundice using a low-cost colour card: a multicentre prospective study. *BMJ Paediatr Open*.

[32] Şahan H, Gülaşı S, Mert MK (2023). The predictive significance of umbilical cord bilirubin and bilirubin/albumin ratio for neonatal jaundice in healthy term newborns. Turk J Med Sci.

[33] Satrom KM, Farouk ZL, Slusher TM (2023). Management challenges in the treatment of severe hyperbilirubinemia in low- and middle-income countries: Encouraging advancements, remaining gaps, and future opportunities. Front Pediatr.

[34] Anderle A, Bancone G, Domingo GJ (2018). Point-of-Care Testing for G6PD Deficiency: Opportunities for Screening. Int J Neonatal Screen.

[35] Watchko JF, Kaplan M, Stark AR (2013). Should we screen newborns for glucose-6-phosphate dehydrogenase deficiency in the United States?. J Perinatol.

