# Supplementary material for: Non-invasive detection of bilirubin concentrations during the first week of life in a low-resource setting along the Thailand–Myanmar border
Source: BMJ Paediatr Open. 2024 Sep 28;8(1):e002754. doi: 10.1136/bmjpo-2024-002754 (PMC11440201; doi:10.1136/bmjpo-2024-002754)
Supplement: online supplemental file 1 [file bmjpo-8-1-s001.pdf]

Supplementary Material

Excluded TcB-TSB paired data points

A total of 6 data points were excluded (circled in blue) from analyses based on the longitudinal data trends as shown in Figure S3.

From the top pane, in the first participant the TSB#4 was probably a reporting mistake; in the second participant, samples #4 and #5 were collected at 5am and 8:40am, we suspect that TSB#4 and TcB#5 could be reporting mistakes. In the third participant, TSB#1 was 35 umol/L with hct=73% while TSB#2 (collected 2hrs later) was 106umol/L with hct=62% therefore we suspected a problem in the sample used for TSB#1 test. In the fourth participant, the two TcB records were exchanged in the logbook but we could not confirm the correct result.

Figure S1. Excluded paired TSB-TcB data points

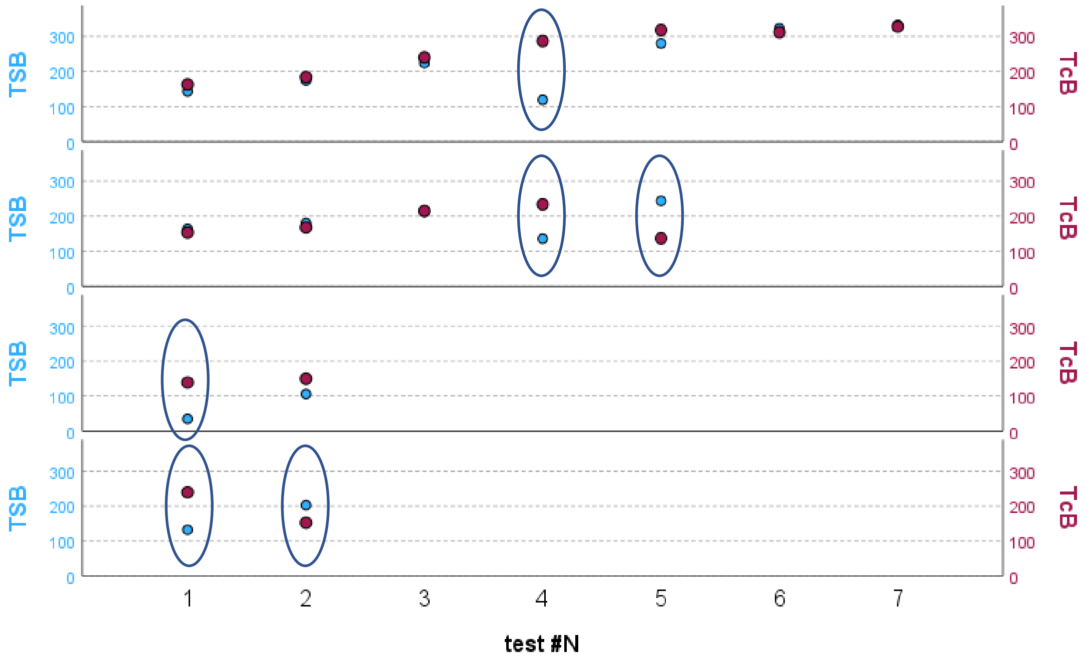

Table S1. Mean difference TcB-TSB for consecutive TSB tests

TSB	N	Mean Difference TcB-TSB	Lower LoA	Upper LoA
1st	296	19.8	-36.4	76.0
2nd	211	25.6	-40.0	91.3
3rd	87	19.5	-48.0	87.0
4th	43	17.8	-59.9	95.5
5th	26	15.8	-42.3	74.0
6th	13	4.5	-55.2	64.1
7th	5	-14.0	-53.7	25.7
8th	3	-34.0	-126.7	58.7
9th	1	0.0		
Total	685	20.5	-42.7	83.6

LoA= Limit of Agreement

Table S2. Sensitivity and specificity of TcB without and with correction at different bilirubin thresholds in all samples

TSB threshold (umol/L)	Sensitivity TcB without correction	Specificity TcB without correction	Sensitivity TcB with correction	Specificity TcB with correction
≥100	0.97	0.50	1.00	0.20
≥150	0.96	0.69	0.99	0.48
≥175	0.94	0.78	0.98	0.64
≥200	0.95	0.84	0.99	0.73
≥225	0.95	0.87	0.96	0.79
≥250	0.88	0.88	0.98	0.83
≥275	0.78	0.93	0.88	0.87
≥300	0.72	0.95	0.90	0.91

Table S3. Performance of the TcB+20 at different bilirubin thresholds by clinic

All clinics	Threshold (umol/L)	N (TcB>= threshold)	Sensitivity	Specificity	PPV	NPV	% of prevented blood tests
	≥100	661	1.00	0.20	0.86	0.92	4
	≥150	517	0.99	0.48	0.65	0.97	25
	≥175	413	0.98	0.64	0.63	0.98	40
	≥200	328	0.99	0.73	0.60	1.00	52
	≥225	250	0.96	0.79	0.55	0.99	64
	≥250	188	0.98	0.83	0.46	1.00	73
	≥275	134	0.88	0.87	0.38	0.99	80
	≥300	84	0.90	0.91	0.31	1.00	88

WPA							
	≥100	235	1.00	0.09	0.83	1.00	2
	≥150	195	0.99	0.32	0.52	0.98	18
	≥175	156	0.99	0.51	0.49	0.99	35
	≥200	117	1.00	0.65	0.43	1.00	51
	≥225	90	1.00	0.71	0.33	1.00	62
	≥250	70	1.00	0.75	0.21	1.00	71
	≥275	50	1.00	0.81	0.12	1.00	79
	≥300	30	NA	NA	NA	NA	
MKT							
	≥100	426	0.99	0.27	0.87	0.91	5
	≥150	322	0.98	0.58	0.72	0.97	28
	≥175	257	0.97	0.72	0.72	0.97	43
	≥200	211	0.99	0.79	0.70	1.00	53
	≥225	160	0.96	0.84	0.67	0.98	64
	≥250	118	0.97	0.88	0.61	0.99	74
	≥275	84	0.87	0.90	0.54	0.98	81
	≥300	54	0.90	0.93	0.48	0.99	88

PPV= positive predictive value; NPV=negative predictive value

Table S4. Performance of corrected TcB thresholds over time

	MKT clinic			WPA clinic								
	2020			2021			2022			2023		
Threshold	N (TcB>= threshold)	se	sp	N (TcB>= threshold)	se	sp	N (TcB>= threshold)	se	sp	N (TcB>= threshold)	se	sp
≥150(umol/L)	321	0.98	0.58	8	1	0.33	147	0.99	0.32	39	1	0.26
≥200(umol/L)	210	0.99	0.79	5	1	0.63	85	1	0.65	26	1	0.62
≥250(umol/L)	117	0.97	0.88	2	1	0.89	49	1	0.77	18	1	0.68
≥300(umol/L)	53	0.89	0.93	2	NA	NA	18	NA	NA	10	NA	NA

NA= not applicable

Figure S2. Examples of longitudinal course of repeated paired TcB-capillary TSB tests in different neonates (1 neonate per pane).

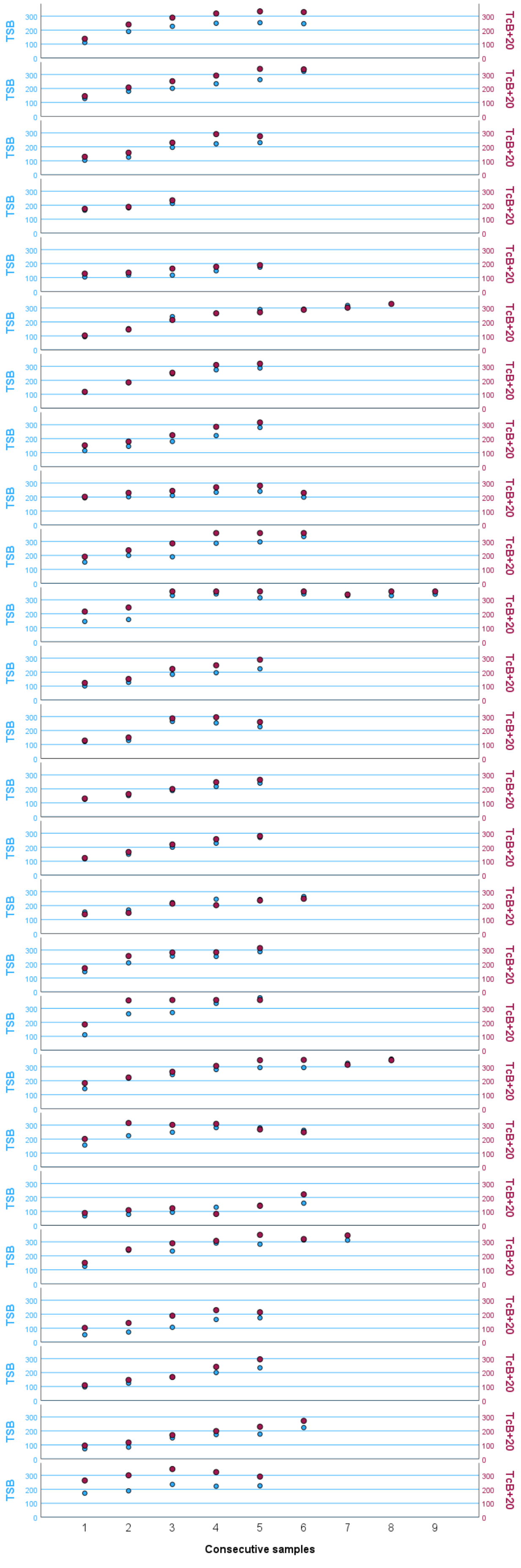

Figure S3. Distribution of TSB in cord blood by clinical site

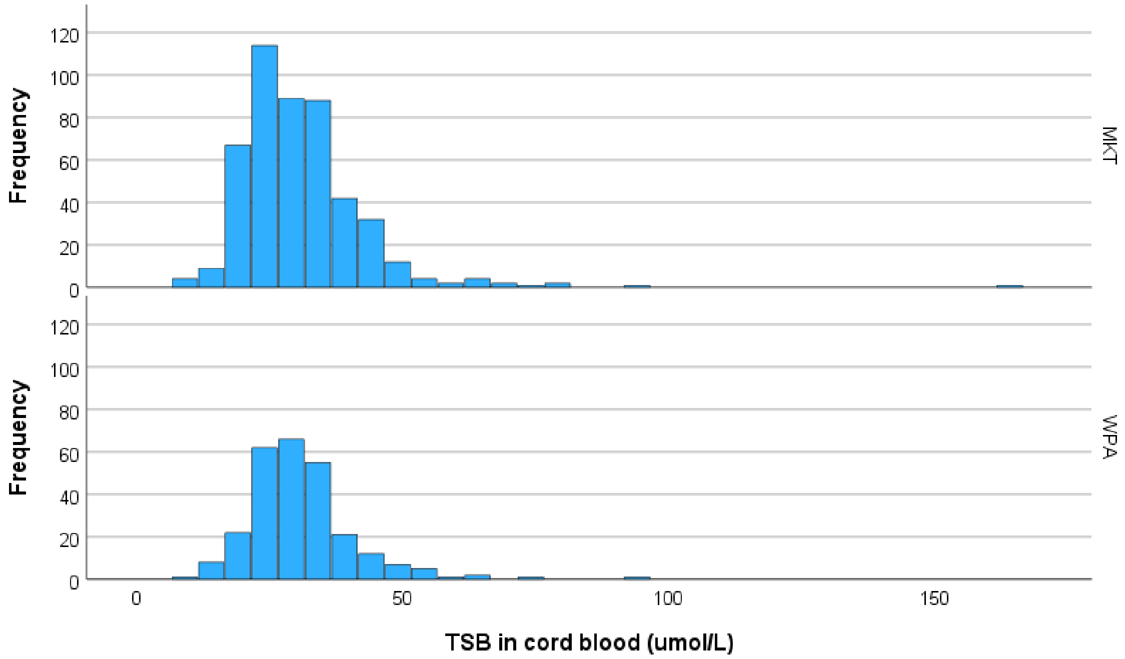

Table S5. Diagnostic performance of cord blood TSB thresholds for identification of NH

Threshold	Population	Reference	Sensitivity	Specificity
>32.0umol/L	All neonates	(1)	67.4%	73.1%
>32.0umol/L	Only EGA≥38 weeks	(1)	77.3%	72.5%
>35.0umol/L	Only EGA≥38 weeks	(2)	71.2%	80.3%

References

1. Guan H, Li H, Luo J, Lin L, Wang Y, Xiao Y, et al. Early predictive value of cord blood bilirubin and dynamic monitoring of transcutaneous bilirubin for hyperbilirubinemia of newborns. Saudi J Biol Sci. 2017;24(8):1879-83.

2. Jones KDJ, Grossman SE, Kumaranayakam D, Rao A, Fegan G, Aladangady N. Umbilical cord bilirubin as a predictor of neonatal jaundice: a retrospective cohort study. BMC pediatrics. 2017;17(1):186.
