# Supplementary material for: Non-invasive detection of bilirubin concentrations during the first week of life in a low-resource setting along the Thailand–Myanmar border
Source: BMJ Paediatr Open. 2024 Sep 28;8(1):e002754. doi: 10.1136/bmjpo-2024-002754 (PMC11440201; doi:10.1136/bmjpo-2024-002754)
Supplement: Supplementary file 2 [file bmjpo-2024-002754.reviewer_comments.pdf]

PEER REVIEW HISTORY

BMJ Paediatrics Open publishes all reviews undertaken for accepted manuscripts. Reviewers are asked to complete a checklist review form and are provided with free text boxes to elaborate on their assessment. These free text comments are reproduced below.

ARTICLE DETAILS

**TITLE (PROVISIONAL)**	Non-invasive detection of bilirubin concentrations during the first week of life in a low-resource setting along the Thailand-Myanmar border
**AUTHORS**	Bancone, Germana; Gilder, Mary; Win, Elsie; Gornsawun, Gornpan; Moo, Paw; Archasuksan, Laypaw; Wai, Nan ; Win, Sylverine; Carrara, Verena; Nosten, Francois; Hanboonkunupakarn, Borimas; McGready, Rose

VERSION 1 - REVIEW

**REVIEWER NAME**	***Bolajoko Olusanya***
**REVIEWER AFFILIATION**	Centre for Healthy Start Initiative, Director
**REVIEWER CONFLICT OF INTEREST**	None
**DATE REVIEW RETURNED**	14-Jun-2024

**GENERAL COMMENTS**	This study: ‘Non-invasive detection of bilirubin concentrations during the first week of life in a low-resource setting along the Thailand-Myanmar border’, is not novel, but non-invasive detection of bilirubin concentration during the first week of life is crucial for newborn care. This is especially important in populations with high rates of G6PD deficiency and in resource-poor communities in low- and middle-income countries where infants often present late with acute bilirubin encephalopathy.<br>The study's results confirmed that cord blood bilirubin is not predictive of hyperbilirubinemia risk in the early days of life. However, it is the first study to report on this issue among neonates in this specific population during the first week of life.<br>The objective of the study is the assessment of a non-invasive transcutaneous bilirubinometer: Dräger Jaundice Meter JM-105, against routine capillary serum bilirubin testing. The methodology appears sound but the presentation and interpretation of the results are problematic.<br>For instance,<br>Page 8, Lines 17-22: ‘Performance of the TcB, calculated as sensitivity and specificity to identify capillary TSB levels over different thresholds, was improved when 20umol/L were added to the reading from the TcB (Table S2), especially at higher thresholds.’<br>The data presented did not show an improvement in specificity; instead, the corrected values decreased. For the effectiveness of screening tests, I will prioritise maintaining specificity over improving sensitivity to reduce the refer rate which has the potential to overburden the already beleaguered system.<br><br>Page 9, Lines 27-32: ‘…TcB values, with an appropriate simple correction, can be used reliably against TSB nomograms to identify

	neonates who need confirmatory laboratory blood tests to detect clinically significant bilirubin levels.'<br>Similar to my earlier comments above, this recommendation is not derived from the data. A robust discussion on the outcome of this correction on the specificity of the test results is critical and should be included in the manuscript.<br><br>Page 9, Lines 48 – 57: 'Even with the low specificity observed especially at 100 or 150umol/L TSB thresholds (which would translate to performing unnecessary blood tests in neonates with true levels of bilirubin lower than those thresholds), the use of TcB would decrease the total number of invasive blood testing required as compared to current practice.'<br>As with my earlier comments, this recommendation is not based on the data. A thorough discussion on the impact of this correction on the specificity compared with the sensitivity is essential and should be included in the discussion. The recommendation will then flow appropriately from the results presented.<br><br>Minor but essential comments:<br>Page 3, Line 12: '…it can pass the blood-brain barrier'<br>Replace 'pass' with 'cross'<br><br>Page 3, Line 39: Define 'HPLC'<br><br>Page 6, Lines 22 - 24: 'Area under the curve (AUC) of the Receiver Operating Characteristic (ROC) curve (17) was calculated for the whole cohort, only for neonates followed-up at least for 48 hours,' Authors, please clarify this statement. Is it referring to the whole population or only neonates followed up for at least 48hours?<br><br>Page 7, Lines 3 -12: 'Within the first 48 hours, 47 neonates completed the study without a capillary TSB or TcB measurement; 43 neonates were admitted to SCBU, 2 were referred to other hospitals and 2 were discharged. In the following 120 hours, the remaining 695 neonates provided 1 to 9 additional capillary blood samples for analysis of TSB at a mean age of 47h for a total of 1244 analyzable samples. Over half of the capillary TSB data point (687 from 307 neonates, 55.2%) had a paired TcB value.'<br>This statement is confusing. Since these 47 babies had no analysable data and were excluded from the analysis, the authors may wish to clarify this statement by replacing 'completed' with 'did not complete'<br><br>Page 7, Lines 15 – 24: The authors need to be consistent with using denominators, percentages or both throughout the manuscript. Do check your figures and percentages.<br><br>Tables & Figures<br>Tables 1& 2 are missing, and the cited Table 3 is the only Table in the manuscript.<br>Table S2 First Column Header: Unit of measurement is missing from TSB threshold

**REVIEWER NAME**	***Vishal Tewari***
**REVIEWER AFFILIATION**	
**REVIEWER CONFLICT OF INTEREST**	None
**DATE REVIEW RETURNED**	29-Jun-2024

GENERAL COMMENTS	Reviewer comments<br><br>Manuscript title: Non-invasive detection of bilirubin concentrations during the first week of life in a low-resource setting along the Thailand-Myanmar border<br><br>Manuscript number: bmjpo-2024-002754<br><br>General comments<br>1. An elegantly conducted study and a very well written manuscript and figures.<br><br>Abstract<br>1. Which capillary microbilirubinometer was used since the TCB device has been mentioned here in the abstract?<br>2. Author states (AS): Bilirubin concentrations in cord blood are not predictive of hyperbilirubinemia in neonates.<br>Reviewer comment (RC): This statement is incorrect. It must be qualified further that "Bilirubin concentrations in cord blood are not predictive of non-immune hyperbilirubinemia in neonates". Since cord blood bilirubin levels may predict the severity of hyperbilirubinemia due to immune causes such as in Rh-isoimmunization.<br><br>Summary box<br>1. What is already known: AS: ….especially in low resource settings.<br>RC: The role of TCB in Low-resource settings and dark skinned neonates is not established. Additionally the cost incurred per test is high as the devices are made by meditech companies in the west.<br>2. What this study adds: AS…..cord blood bilirubin levels are not predictive of hyperbilirubinaemia risk in the first days of life.<br>RC: Please see my comment no. 2 for the Abstract section given above.<br><br>Introduction<br>1. Page 3, Line 18: AS: Several risk factors…<br>RC: The list of risk factors must mention Rh incompatibility as it continues to be an important cause of severe neonatal jaundice in LMIC setting.<br><br>Material and methods<br>1. What were the performance characteristics of the Capillary microbilirubinometer as mentioned by the manufacturer? Was it a single-beam or twin-beam device and its performance at higher ranges of Total Bilirubin such as 300-500 micromol/L compared with the Transcutaneous device. This information is important since essentially the study is testing a TCB device against the Capillary bilirubinometer.<br>2. The study is silent about Rh-isoimmunization in the study population. Is it not a problem of public health importance in the region where this study was done?<br><br>Results<br>1. Page 8, line 20: AS: ….different thresholds, was improved when 20umol/L were added to the reading from the TcB….<br>RC: What is the basis of this applied correction? Is it a manufacturers recommendation? Why 20 and why not 10 or 30? It

	would certainly vary between devices of different manufacturers seriously affecting the external validity of the study.<br>2. Page 10, line 43: AS…They were higher in neonates born with EGA 35 to < 38….<br>RC: This must be explained in the discussion section.<br>3. Page 10, line 48: AS…Statistically higher mean (SD) cord TSB [41.0 (17.6) umol/L] was observed….<br>RC: With this result why are authors stating that cord Bilirubin levels were not predictive of the need for phototherapy. The ROC may not be able to identify a cut-off predictive of NH requiring treatment but cord TSB was significantly higher in the group which required treatment later.<br><br>Discussion<br>1. Page 11, line 26-27: AS….capillary TSB levels in neonates of Karen and Burman ethnicity here for the first time.<br>RC: Ethnicity should not have any effect on the applicability of Transcutaneous bilirubinometry, other than the limitation in neonates with dark skin which has already been mentioned. The authors may want to reconsider this statement.<br>2. Page 11, line 28: AS….appropriate simple correction…<br>RC: How did the authors decide on this appropriate simple correction? Please see comment no. 1 in Results section.<br>3. The addition of the Transcutaneous bilirubinometer will not result in removal of the Capillary microbilirubinometer (TCB are not accurate at values above 300 micromol/L (18 mg/dl) and once phototherapy has been instituted). Thus need for more than 2 technologies to be maintained (Transcutaneous bilirubinometer, Capillary microbilirubinometer, and autoanalyser for lab estimation of Total bilirubin) is actually not tenable in most LMIC settings.

VERSION 1 – AUTHOR RESPONSE

Reviewer: 1

Dr. Bolajoko Olusanya, Centre for Healthy Start Initiative

Comments to the Author

Q#1. Page 8, Lines 17-22: 'Performance of the TcB, calculated as sensitivity and specificity to identify capillary TSB levels over different thresholds, was improved when 20umol/L were added to the reading from the TcB (Table S2), especially at higher thresholds.'

The data presented did not show an improvement in specificity; instead, the corrected values decreased. For the effectiveness of screening tests, I will prioritise maintaining specificity over improving sensitivity to reduce the refer rate which has the potential to overburden the already beleaguered system.

R#1. We appreciate this comment and agree that lower specificity is associated with performing unnecessary blood testing in neonates with lower bilirubin concentrations. However, in this low-resource setting with highly mobile population and difficult to reach health facilities, our primary aim would be to minimize the risk of missing high bilirubin concentrations; for this reason, we would aim for higher sensitivity in a screening test. We have added more about this in the results and in the discussion and mentioned that this approach might limit generalizability of the findings.

Q#2. Page 9, Lines 27-32: '…TcB values, with an appropriate simple correction, can be used reliably against TSB nomograms to identify neonates who need confirmatory laboratory blood tests to detect clinically significant bilirubin levels.'

Similar to my earlier comments above, this recommendation is not derived from the data. A robust discussion on the outcome of this correction on the specificity of the test results is critical and should be included in the manuscript.

R#2. We have added more explanation on the rationale for prioritizing higher sensitivity at the expenses of specificity. See also R#8 to reviewer 2

Q#3. Page 9, Lines 48 – 57: 'Even with the low specificity observed especially at 100 or 150umol/L TSB thresholds (which would translate to performing unnecessary blood tests in neonates with true levels of bilirubin lower than those thresholds), the use of TcB would decrease the total number of invasive blood testing required as compared to current practice.'

As with my earlier comments, this recommendation is not based on the data. A thorough discussion on the impact of this correction on the specificity compared with the sensitivity is essential and should be included in the discussion. The recommendation will then flow appropriately from the results presented.

R#3. We have explained better what is the current practice in our context and revise the text accordingly. We have added the percentage of blood confirmatory tests that would be prevented in TcB was used as pre-discharge screening in our clinical setting.

Q#4. Minor but essential comments:

Page 3, Line 12: '…it can pass the blood-brain barrier'

Replace 'pass' with 'cross'

R#4. This has been amended

Q#5. Page 3, Line 39: Define 'HPLC'

R#5. This has been amended

Q#6. Page 6, Lines 22 - 24: 'Area under the curve (AUC) of the Receiver Operating Characteristic (ROC) curve (17) was calculated for the whole cohort, only for neonates followed-up at least for 48 hours,'

Authors, please clarify this statement. Is it referring to the whole population or only neonates followed up for at least 48hours?

R#6. This sentence is listing the 3 groups on which we run AUC of ROC. It has been changed to: "'Area under the curve (AUC) of the Receiver Operating Characteristic (ROC) curve (17) was

calculated for 1) the whole cohort, 2) for neonates followed-up for at least 48 hours, and 3) at different cord blood TSB thresholds'

Q#7. Page 7, Lines 3 -12: 'Within the first 48 hours, 47 neonates completed the study without a capillary TSB or TcB measurement; 43 neonates were admitted to SCBU, 2 were referred to other hospitals and 2 were discharged. In the following 120 hours, the remaining 695 neonates provided 1 to 9 additional capillary blood samples for analysis of TSB at a mean age of 47h for a total of 1244 analyzable samples. Over half of the capillary TSB data point (687 from 307 neonates, 55.2%) had a paired TcB value.'

This statement is confusing. Since these 47 babies had no analysable data and were excluded from the analysis, the authors may wish to clarify this statement by replacing 'completed' with 'did not complete'

R#7. We have clarified the statement and have corrected the text. It now reads; "Within the first 48 hours, 47 neonates with cord blood TSB results exited the study without a capillary TSB or TcB measurement; of these 47 neonates, 43 were admitted to SCBU, 2 were referred to other hospitals and 2 were discharged"

Q#8. Page 7, Lines 15 – 24: The authors need to be consistent with using denominators, percentages or both throughout the manuscript. Do check your figures and percentages.

R#8. We have checked figures and text throughout the manuscript

Q#9. Tables & Figures

Tables 1& 2 are missing, and the cited Table 3 is the only Table in the manuscript.

Table S2 First Column Header: Unit of measurement is missing from TSB threshold

R#9. Yes, Tables were moved to supplementary material on the last revision of the manuscript and numbering was not updated, thanks for spotting this! We have corrected this and added the missing units in Table S2 and S4.

Reviewer: 2

Dr. Vishal Tewari

Comments to the Author

Q#1. Which capillary microbilirubinometer was used since the TCB device has been mentioned here in the abstract?

R#1. We have now included this in the abstract.

Q#2. Author states (AS): Bilirubin concentrations in cord blood are not predictive of hyperbilirubinemia in neonates.

Reviewer comment (RC): This statement is incorrect. It must be qualified further that “Bilirubin concentrations in cord blood are not predictive of non-immune hyperbilirubinemia in neonates”. Since cord blood bilirubin levels may predict the severity of hyperbilirubinemia due to immune causes such as in Rh-isoimmunization.

R#2. In this work, cord blood bilirubin levels were analysed with respect of phototherapy treatment for hyperbilirubinaemia of any origin; therefore, we feel it would be more accurate to keep the sentence as it is now.

Summary box

Q#3. What is already known: AS: ….especially in low resource settings.

RC: The role of TCB in Low-resource settings and dark skinned neonates is not established. Additionally the cost incurred per test is high as the devices are made by meditech companies in the west.

R#3. We have included the dark skinned info in the text of the summary box. Since we have not made a detailed cost analysis of the TcB in this work, we would like to avoid including this in the summary box. We have discussed the issue in the discussion.

Q#4. What this study adds: AS…..cord blood bilirubin levels are not predictive of hyperbilirubinaemia risk in the first days of life.

RC: Please see my comment no. 2 for the Abstract section given above.

R#4. Please see our answer to Q#2

Introduction

Q#5. Page 3, Line 18: AS: Several risk factors…

RC: The list of risk factors must mention Rh incompatibility as it continues to be an important cause of severe neonatal jaundice in LMIC setting.

R#5. We have added it.

Material and methods

Q#6. What were the performance characteristics of the Capillary microbilirubinometer as mentioned by the manufacturer? Was it a single-beam or twin-beam device and its performance at higher ranges of Total Bilirubin such as 300-500 micromol/L compared with the Transcutaneous device. This information is important since essentially the study is testing a TCB device against the Capillary bilirubinometer.

R#6. We agree this is an important information and it was already reported in the materials and methods section. We have added the range for the units used in the study. “The BR-501 bilirubinometer had ± 5% accuracy within the measurement range (0–30 mg/dl or 0-513 umol/L), as reported by the manufacturer.”

Q#7. The study is silent about Rh-isoimmunization in the study population. Is it not a problem of public health importance in the region where this study was done?

R#7. This is correct, Rh incompatibility is very low in Asia; in the population attending SMRU clinics the prevalence of Rh- group is <0.2%.

Results

Q#8. Page 8, line 20: AS: ….different thresholds, was improved when 20umol/L were added to the reading from the TcB….

RC: What is the basis of this applied correction? Is it a manufacturers recommendation? Why 20 and why not 10 or 30? It would certainly vary between devices of different manufacturers seriously affecting the external validity of the study.

R#8. The correction was found empirically with the aim of increasing the test’s sensitivity to 98% for most of the thresholds. We agree that using the correction might decrease the generalizability of this finding; nevertheless, we have presented the performance of the test using the un-corrected TcB values (Supplementary Table 2) and we leave to clinicians and researchers to apply the correction that is most suitable for their specific context. We have added this in the limitations of the study.

Q#9. Page 10, line 43: AS…They were higher in neonates born with EGA 35 to < 38….

RC: This must be explained in the discussion section.

R#9. We have added this sentence in the discussion. “This analysis of bilirubin concentrations in cord blood showed higher mean TSB in premature neonates and in neonates who needed phototherapy during the first week of life but found no meaningful thresholds for identification of neonates at risk of NH, in all neonates or in the sub-group of term neonates.

Q#10. Page 10, line 48: AS…Statistically higher mean (SD) cord TSB [41.0 (17.6) umol/L] was observed….

RC: With this result why are authors stating that cord Bilirubin levels were not predictive of the need for phototherapy. The ROC may not be able to identify a cut-off predictive of NH requiring treatment but cord TSB was significantly higher in the group which required treatment later.

R#10. Despite a statistically significant higher mean cord TSB in the jaundiced group, due to the distribution of cord TSB results, the individual cord blood TSB cannot be used reliably to predict need of treatment. For instance, among neonates with lower cord TSB (<32umol/L), over 30% needed phototherapy. Only among neonates with cord TSB <21umol/L there was no need of phototherapy; nevertheless, they represented a very small proportion (13%) of the neonatal population while for the majority of neonates the cord TSB concentration does not provide useful information.

Discussion

Q#11. Page 11, line 26-27: AS….capillary TSB levels in neonates of Karen and Burman ethnicity here for the first time.

RC: Ethnicity should not have any effect on the applicability of Transcutaneous bilirubinometry, other than the limitation in neonates with dark skin which has already been mentioned. The authors may want to reconsider this statement.

R#11. We have modified the sentence.

Q#12. Page 11, line 28: AS….appropriate simple correction…

RC: How did the authors decide on this appropriate simple correction? Please see comment no. 1 in Results section.

R#12.Please see answer to Q#8

Q#13. The addition of the Transcutaneous bilirubinometer will not result in removal of the Capillary microbilirubinometer (TCB are not accurate at values above 300 micromol/L (18 mg/dl) and once phototherapy has been instituted). Thus need for more than 2 technologies to be maintained (Transcutaneous bilirubinometer, Capillary microbilirubinometer, and autoanalyser for lab estimation of Total bilirubin) is actually not tenable in most LMIC settings.

R#13. We agree with the reviewer that the TcB will not replace the reference test on blood; however, there are several scenarios for use of the TcB as a screening tool both before and during phototherapy (the latest version of the Drager JM-105 has been evaluated in neonates undergoing phototherapy with good results), to increase repeated pain-free testing in at risk neonates and to be used in those settings where no laboratory tests are available. We agree that using several technologies in low-resource settings brings specific issues and does require more efforts and QC/QA system which are not always easy in LMIC settings.

VERSION 2 – REVIEW

**REVIEWER NAME**	***Bolajoko Olusanya***
**REVIEWER AFFILIATION**	Centre for Healthy Start Initiative, Director
**REVIEWER CONFLICT OF INTEREST**	None
**DATE REVIEW RETURNED**	19-Jul-2024

**GENERAL COMMENTS**	Unable to complete the review with this submission. Authors need to provide detailed rebuttals with page and line numbers to my earlier comments and submit a version with corresponding track changes to the original manuscript.

**REVIEWER NAME**	***Vishal Tewari***
**REVIEWER AFFILIATION**	
**REVIEWER CONFLICT OF INTEREST**	None

**DATE REVIEW RETURNED**	21-Jul-2024

**GENERAL COMMENTS**	Reviewer comments<br><br>Manuscript title: Non-invasive detection of bilirubin concentrations during the first week of life in a low-resource setting along the Thailand-Myanmar border<br><br>Manuscript number: bmjpo-2024-002754.R1<br><br>1. The low prevalence of Rh-incompatibility in the specific ethnic population is required to be mentioned in the manuscript. Rh-incompatibility continues to be a major public health problem in many LMIC countries in Asia.<br>2. Authors comments about TcB not replacing capillary microbilirubinometer and the difficulties in maintaining multiple technologies must be added as a limitation of this study.

VERSION 2 – AUTHOR RESPONSE

Reviewer: 2

Dr. Vishal Tewari

Comments to the Author

Q#1. The low prevalence of Rh-incompatibility in the specific ethnic population is required to be mentioned in the manuscript. Rh-incompatibility continues to be a major public health problem in many LMIC countries in Asia.

We acknowledge the reviewer's concern about Rh-incompatibility being a major public health problem. SMRU introduced Rh testing in 2014 and in a longitudinal birth cohort study on the epidemiology of neonatal jaundice there was a very low rate of Rhesus incompatibility 3/1700 (0.2) [https://doi.org/10.1371/journal.pone.0258127] and this detail is now incorporated into the manuscript. See line 61 where Rh-incompatibility is added as a general risk factor, and line 96 as incidence.

Q#2. Authors comments about TcB not replacing capillary microbilirubinometer and the difficulties in maintaining multiple technologies must be added as a limitation of this study.

R#2. We have to disagree with this statement as the TcB is, by definition, a screening test and its scope of use is not to replace microbilirunometric test on blood. We have however added this sentence at the end of the discussion (line 268) "Maintaining multiple technologies and providing appropriate QA/QC in low-resource settings can be problematic." to reflect the reviewer suggestion and the comments we made in the previous revision.

VERSION 3 – REVIEW

**REVIEWER NAME**	***Bolajoko Olusanya***
**REVIEWER AFFILIATION**	Centre for Healthy Start Initiative, Director
**REVIEWER CONFLICT OF INTEREST**	None
**DATE REVIEW RETURNED**	03-Sep-2024

**GENERAL COMMENTS**	All my concerns have been addressed satisfactorily. No further comments.

**REVIEWER NAME**	***Vishal Tewari***
**REVIEWER AFFILIATION**	
**REVIEWER CONFLICT OF INTEREST**	None
**DATE REVIEW RETURNED**	08-Sep-2024

**GENERAL COMMENTS**	The authors have suitably answered the queries raised in the previous reviews.
